# Pediatric fluid resuscitation: an oxymoron?

**DOI:** 10.3389/fped.2025.1594336

**Published:** 2025-06-10

**Authors:** Franco Diaz, Jocelyn Riderelli, Roberto Jabornisky

**Affiliations:** ^1^ Unidad de Paciente Crítico Pediátrico, Hospital El Carmen de Maipú, Santiago, Chile; ^2^ Unidad de Investigación y Epidemiología Clínica (UIEC), Escuela de Medicina, Universidad Finis Terrae, Santiago, Chile; ^3^ Red Colaborativa Pediatrica de Latinoamérica, LARed Network, Santiago, Chile; ^4^ Unidad de Cuidados Intensivos Pediátricos, Hospital Regional Olga Stucky de Rizzi, Reconquista, Argentina

**Keywords:** sepsis, fluid over load, septic shock, pediatric, fluids

Fluid administration has been a cornerstone of therapy for acutely ill children for decades. The association between inadequate hydration, diarrhea, and dehydration with disease severity—and ultimately, mortality— in children has been acknowledged since antiquity. Despite significant advancements in healthcare that have reduced infant mortality rates, like oral rehydration therapy promoted by the World Health Organization, approximately 500,000 children continue to die each year from diarrhea and dehydration, primarily in low-resource settings (1).

Since the 1990s, research has highlighted key differences in how children and adults respond to severe infections, particularly in hemodynamics and organ failure patterns. These differences have led to the belief that hypovolemia is the main hemodynamic issue in children with severe infections. Unsurprisingly, as fast as intravenous fluids were pushed, we rushed to conclude that promptly correcting hypovolemia could prevent disease progression and improve outcomes. This approach received endorsement from numerous academic societies and was precipitously integrated into sepsis and septic shock treatment protocols. It became widely adopted globally as a “one-size-fits-all” recommendation, particularly due to the easy availability of fluids such as normal saline and Ringer's lactate.

However, early studies advocating for “*aggressive fluid resuscitation*” were limited in scope. A seminal study published in JAMA in 1991, which analyzed 34 children, indicated improved survival rates with increased fluid administration (greater than 40 ml/kg) (2). Yet, this study reported that 82% of patients required invasive mechanical ventilation within six hours, and five children developed pulmonary edema. In high-income countries, the detrimental effects of “*aggressive fluid resuscitation*” could be easily monitored (pulse oximetry, blood gas analysis, chest x-ray) and promptly treated with oxygen, as well as non-invasive and invasive mechanical ventilation. It was reasonable to recommend a potentially lifesaving therapy despite the possibility of adverse effects, as the benefits of preventing death outweighed the risks of such outcomes if treated accordingly (3).

Over time, accumulating evidence has highlighted the harmful effects of rapid, large-volume fluid administration, particularly in resource-limited environments where managing complications is challenging (4, 5). It has taken over two decades for the medical community to cautiously acknowledge these adverse effects, with recommendations to reduce initial fluid boluses; however, these changes have not yet significantly influenced clinical practice (5–10).

Young children are particularly susceptible to dehydration due to their physiological characteristics, including a higher body water percentage, increased metabolic rate, and renal immaturity. Clinical confusion often arises between dehydration and hypovolemia, leading to sub-optimal therapy. Dehydration refers to a deficit in total body water and is usually associated with hypertonicity, whereas hypovolemia refers to a reduction in circulating blood volume, about 10% of total body water. Pediatric patients can experience dehydration without significant hypovolemia, as fluid may redistribute to maintain blood volume. It is important to recall that between 60% and 70% of the intravascular water is contained in the highly compliant venous central compartment, also known as non-stressed volemia according to Guyton's model of the circulatory system. Rapid administration of intravenous fluids in large quantities will transiently increase hydrostatic pressure in the venous compartment but ultimately will end up in the non-stressed compartment rather than a sustained hemodynamic improvement, particularly when the venous tone remains unchanged (11, 12).

Criticism of fluid resuscitation therapy has increased as evidence of its potential harms grows, prompting a call to rationalize individualized therapy (13, 14). Nonetheless, rehydration and the correction of hypovolemia remain crucial interventions for children, saving countless lives. Unfortunately, in a classic egocentric bias, pediatric critical care guidelines have not adequately incorporated personalized medicine principles, particularly regarding pathophysiology and the diverse contexts in which pediatric patients receive care. Notably, 86% of the authors of the guidelines are from high-income countries (3, 5).

Healthcare professionals must recognize the cognitive biases that may have led to overestimating the benefits of fluid resuscitation, especially in sepsis protocols. Thousands of children receive unnecessary fluid boluses, which can contribute to increased morbidity and mortality. Recent initiatives, such as the Phoenix criteria for sepsis, aim to refine patient selection for timely interventions, but further research is required to validate this approach compared to traditional methods (15).

Given these considerations, we should have emphasized the potential adverse effects of rapid fluid administration. Like most treatments for critically ill children, healthcare professionals must be alert to common complications and know how to manage them. Second, it is essential to highlight that not all hemodynamically unstable children suffer from severe hypovolemia requiring the rapid infusion of nearly one blood volume (60 ml/kg) within minutes (16, 17). Third, a reduction in stressed circulating volume contributes to poor organ perfusion, making the early initiation of vasoactive drugs a key intervention. Notably, starting epinephrine or norepinephrine via peripheral venous access is safe (18, 19). Fourth, the growing population of technology-dependent children, with complex-chronic-conditions and with central venous catheters, frequently present with low systemic vascular resistance often present with low systemic vascular resistance, a distinctive hemodynamic profile (20). Recent studies show this population may account for up to 50% of PICU admissions due to sepsis, even in developing countries. Fifth, the risk of adverse effects from rapid intravenous fluid boluses increases when administered quickly, often leading to a higher incidence of respiratory failure within the first hour, requiring mechanical ventilation (21). Sixth, the hemodynamic benefits of rapid fluid infusion are transient, peaking within the first 5 min, with most cardiovascular effects diminishing after 10 min and disappearing within an hour. Increased hydrostatic pressure can lead to interstitial fluid accumulation and exacerbate capillary leak through glycocalyx damage and endothelial injury. Additionally, fluid boluses are often given after the first 6 h of hospital admission and during the second or third day in the PICU, driven by outdated views that dismiss edema as a mere cosmetic issue or assume “no congestion, no harm”. (22, 23) These practices contribute to electrolyte imbalances, fluid overload, and ICU-acquired morbidity (24).

The term “oxymoron” refers to a phrase that combines contradictory elements, such as “awfully good”. Resuscitation denotes the act of restoring life to an individual and, in critical care, is correcting physiological disorders in acutely ill patients. Given the current evidence, the term “fluid resuscitation” appears contradictory. Looking again at the definition, isn't it curious that we must “*de-resuscitate*” a patient? The concept of “de-resuscitation”, where excess fluid is later removed, further underscores the paradox inherent in current fluid management practices.

Given these insights, we must question: Are we genuinely *resuscitating* patients with fluids? From our perspective, the phrase’ fluid resuscitation' itself is an oxymoron. We must redefine fluid management terminologies—shifting from “resuscitation” to more precise terms, clarifying its purpose: rehydration, replacement of losses, maintenance, treatments, nutrition, and monitoring (25).

In conclusion, fluid therapy is vital for caring for critically ill children, but the rationale for employing fluid boluses as a blind blanket treatment for hemodynamic instability requires thorough reassessment. A comprehensive understanding of fluids as a drug in critical care, including a dose, duration, and de-escalation (4D's defined by Malbrain et al.), is essential, with specific indications and duration (25). A more rational, individualized approach may facilitate the adoption of alternative strategies aimed at minimizing excessive fluid administration and preventing fluid overload (26). As underscored by Fernandez-Sarmiento et al. (13), it is imperative that clinicians systematically assess and clearly document, at least on a daily basis, the current phase of critical illness in each pediatric patient to ensure fluid management is appropriately tailored. Given the mounting evidence highlighting the potential harms and only transient benefits of fluid resuscitation, a precise, individualized, and context-driven approach to fluid therapy is no longer optional—it is essential. Such a strategy is crucial for optimizing outcomes and minimizing iatrogenic complications in critically ill children globally.
